# Herbal Formula Longteng Decoction Promotes the Regression of Synovial Inflammation in Collagen-Induced Arthritis Mice by Regulating Type 2 Innate Lymphocytes

**DOI:** 10.3389/fphar.2021.778845

**Published:** 2021-12-23

**Authors:** Huijie Zhang, Juan Liu, Pingxin Zhang, Dongyang Li, Guiyu Feng, Meiyier Huandike, Song Sun, Limin Chai, Jingwei Zhou

**Affiliations:** ^1^ Key Laboratory of Chinese Internal Medicine of Ministry of Education and Beijing, Dongzhimen Hospital, Beijing University of Chinese Medicine, Beijing, China; ^2^ Department of Rheumatology, Dongzhimen Hospital, Beijing University of Chinese Medicine, Beijing, China

**Keywords:** Longteng Decoction, rheumatoid arthritis, type 2 innate lymphocytes, synovial inflammation, STAT6 signal pathway

## Abstract

The etiology and pathogenesis of rheumatoid arthritis (RA) have not yet been fully elucidated, with greater adverse drug effects in traditional treatment of RA. It is particularly necessary to develop and study Chinese herbal formula as a supplement and alternative drug for the treatment of RA. The traditional Chinese medicine compound Longteng Decoction (LTD), as an empirical prescription in the treatment of RA in Dongzhimen Hospital of Beijing University of Chinese Medicine, has been widely used in clinic. Type 2 innate lymphocytes (ILC2s) have specific transcription factors and signature cytokines that are very similar to Th cells, which have been proved to be necessary in addressing RA inflammation, and are potential targets for RA prevention and treatment. Our previous studies have confirmed that LTD can intervene in the differentiation of peripheral blood Th17 and Treg cells, reduce joint pain index and swelling degree, shorten the time of morning stiffness, reduce ESR, and inhibit joint inflammation. However, it is unclear whether LTD can promote the regression of RA synovial inflammation by regulating the immune response mechanism of ILC2s.Therefore, our team established a collagen-induced arthritis mouse model and conducted an experimental study with LTD as the intervention object. The results showed that joint swelling, synovial inflammatory infiltration, and articular cartilage destruction were alleviated in CIA mice after intervention with LTD. The proliferation and differentiation of Th17 inflammatory cells and the secretion of proinflammatory cytokines (IL-17 and IFN-γ) were inhibited. In addition, LTD can also activate ILC2s to secrete the anti-inflammatory cytokine IL-4, activate the STAT6 signaling pathway, and act synergistic with Treg cells to inhibit the infiltration of type M1 macrophages in synovial tissue and promote its transformation to M2 phenotype. Taken together, these results confirm that LTD can be used as an adjunct or alternative to RA therapy by modulating the ILC2s immune response network and slowing down the inflammatory process of synovial tissue.

## Introduction

Rheumatoid arthritis (RA) is a general chronic autoimmune disease with an incidence rate of 0.4–1.3% (Lin et al., 2020). The disease mainly invades synovial and joints, and its pathological manifestations include synovial hyperplasia with extensive inflammatory cells infiltration, the generation of pannus and the damage of articular cartilage, with a high disability rate (Daniel and Josef, 2018; Stefan et al., 2015). Although the detailed pathogenesis of RA are still unclear, synovial inflammation remains a major target of the disease in its mature clinical stage (Bugatti et al., 2019). Synovitis is an inflammation of the joint capsule composed of synovium, synovial fluid, and their bones (Daniel and Josef, 2018). During the development of RA, synovial membranes undergo the proliferation and activation of different immune cells, as well as inflammatory changes such as innate and adaptive immune cells infiltration (Firestein and McInnes, 2017; Orr et al., 2017). The constant immune cell activation leads to chronic inflammatory of the joints and synovial swelling, which in turn leads to synovial expansion and invasion of the periarticular bone at the cartilage-bone junction, leading to cartilage degeneration and bone invasion (Daniel and Josef, 2018).

Activation of innate immunity may be a key pathogenic mechanism of synovial inflammation (Firestein, 2003). Innate lymphocytes (ILCs) are the newly found immune cells that are not antigen-specific. It is divided into three different subunits, ILC1s, ILC2s, and ILC3s (Shikhagaie et al., 2017). It has specific transcription factors and signature cytokines that are very similar to Th cells (Yasunori et al., 2018; Guia and Narni-Mancinelli, 2020), and has been considered as an essential component of innate immunity in immune arthritis and plays an important role in recent years (MA et al., 2017; Rauber et al., 2017). Abnormal activation of ILCs (except NKs) has been proved to be a pathogenic factor of RA (Edilova et al., 2020), where ILC2s are essential and play an important role in addressing RA inflammation (Yamada et al., 2007). ILC2s are proliferated and activated after co-stimulation by IL-33 and IL-9, secreting anti-inflammatory cytokines IL-14 and IL-4, activating the STAT6 signaling pathway, and inducing macrophages to convert to M2 phenotype (Chen et al., 2019; Schmitz et al., 2005; Yasunori et al., 2018). Furthermore, it plays a synergistic effect with Treg cells in pathological tissues to inhibit the infiltration of M1 macrophages and neutrophils in synovial tissues, proliferation of Th17 cells and secretion of IL-17 (pro-inflammatory cytokine), repair the inflammatory damage of articular cartilage, slow down the inflammatory process of synovial tissues, and promote the alleviation and regression of joint inflammation (Rauber et al., 2017).

The JAK/STAT signaling pathway can promote synovial hyperplasia and play a key role in the pathogenesis of RA synovitis (Ptacek et al., 2021). Studies have suggested that JAK2-STAT1/3 may be the upstream mechanism of FLS proliferation inhibition (Tamai et al., 2006). Previous studies also reported the expression of activated STAT6 and JAK3 in RA synovial tissue (Wang et al., 2012; Walker et al., 2006). STAT6 can be activated by IL-4 and IL-13, mainly regulates Th2 differentiation, and inhibits arthritis and inhibits osteoclast differentiation (Omata et al., 2020; Omata et al., 2018). At present, JAK2 and JAK3 targeting inhibitors have been widely used in clinical treatment, but their side effects are relatively large (Lin et al., 2020). The natural ingredients in Traditional Chinese medicine have been proved to have good effects in targeting JAK2 and JAK3 to improve inflammation and arthritis (Wang et al., 2020). Therefore, exploring the regulatory effect of traditional Chinese medicine on the JAK/STAT signaling pathway may provide new strategies and new targets for the treatment of RA.

Longteng Decoction (LTD) is a Chinese herbal formula based on the theory of “healthy qi deficiency, latent pathogenic obstruction” of Chinese medicine. It is also an empirical formula for RA treatment in Dongzhimen Hospital of Beijing University of Chinese Medicine, and has been widely used in clinical practice. LTD consists of six important components: Dioscorea nipponica, Honeysuckle stem, Sinomenium acutum, Paeonia lactiflora Pall, Artemisia annua, and Trachelospermum jasminoides. It was shown that the effective component of Dioscorea nipponica total saponins can inhibit RA synovial inflammation by inhibiting the expression of nuclear transcription factors in fibroblast-like synovial cells, reducing synovial tissue hyperplasia and inflammatory cell infiltration (Duan et al., 2014). Moreover, it can also alleviate collagen-induced arthritis by inhibiting the Th17 cells response (Li and Zhou, 2020). In previous experiments, we also confirmed that chlorogenic acid and luteolin, the major ingredients of Honeysuckle stem, can inhibit FLSs inflammatory proliferation (Li et al., 2017; Nie et al., 2016; Lou et al., 2016; Lou et al., 2015). Our clinical research has also shown that (Wang Y. et al., 2021; Chen et al., 2019) LTD can reduce joint pain index and swelling degree, shorten morning stiffness time, inhibit disease progression, reduce ESR, and improve patients’ quality of life. LTD can also interfere with the proliferation of Th17 and Treg cells in peripheral blood, and significantly improve hemorheology related indicators. Although LTD is well effective in the treatment of RA, there is a lack of basic experimental studies. And the specific mechanism of its action in regulating the immune response network and promoting the regression of synovial inflammation is still unclear.

Our research group found that ILC2s is a potential target for RA prevention and treatment. ILCs have specific transcription factors and signature cytokines extremely similar to Th cells. Our previous studies have demonstrated that LTD can interfere with the differentiation of Th cells. Therefore, we proposed a hypothesis that LTD can inhibit abnormal immune response, maintain environmental immune homeostasis in pathological tissues, and slow down the inflammatory process of synovial tissues by regulating the ILC2s immune response network, which is the key effecting mechanism in the treatment of RA inflammation. In this research, collagen-induced CIA mice as an animal model and LTD was used as an intervention measure to explore the possible mechanism of LTD regulating ILC2s, providing evidence for LTD as an effective herbal formula for RA treatment.

## Materials and Methods

### Preparation of the Herbal Medicine and HPLC-ESI/MSn Analysis

LTD comprises six herbs: 50 g Chuan Shanlong (Dioscorea nipponica), 30 g Ren Dongteng (Honeysuckle stem), 25 g Qing Fengteng (Sinomenium acutum), 25 g Sheng Baishao (Paeonia lactiflora Pall), 25 g Qing Hao (Artemisia annua), 30 g Luo Shiteng (Trachelospermum jasminoides). The herbs were provided by the Pharmacy Department of Dongzhimen Hospital of Beijing University of Chinese Medicine. After decocting in water, they were heated and concentrated. And the liquid was stored in a refrigerator at 4°C for later use.

Based on the method we described earlier, the composition of LTD was determined using HPLC-ESI/MSn (Li et al., 2017). The TripleTOF™ 5600 Liquid Chromatography High Resolution Tandem Mass Spectrometer (SCIEX, United States) was used to analyze the data including signal intensity and retention time.

### Animal Groups and Treatments

A total of 40 DBA/1 male mice aged 7–8 weeks (weight 18 ± 2 g) were purchased from Beijing Weitong Lihua Laboratory Animal Co., Ltd. (Beijing, China). All mice were maintained in a specific pathogen–free animal facility at the Dongzhimen Hospital of Beijing University of Chinese Medicine and allowed access to food and water ad libitum. This study was approved by the Animal Ethics Committee of the Beijing University of Chinese Medicine and the management and use requirements of the experimental animal ethics committee of Dongzhimen Hospital of Beijing University of Traditional Chinese Medicine were strictly followed during the experiment. After 7 days of adaptive feeding, 8 mice were randomly selected as the blank group and the remaining 32 mice were used for CIA modeling.

CIA induction was performed as previously described (Deng et al., 2012). Briefly, it was emulsified with 100 μg of bovine type II collagen (CII) and the same amount of complete Freund’s adjuvant (CFA), and immunization was given to the base of the tail of the mouse. Then, a boost immunization of CII emulsified in incomplete Freund’s adjuvant (IFA) was carried out on the 21st day after the first immunization. A total of 28 mice were successfully modeled (incidence rate 87.5%). After the successful establishment of the model, 24 mice were freely divided into model group, LEF group, and LTD group (*n* = 8 mice/group). Mice were treated with drug intervention after 7 days of enhanced immunity. The dose was estimated based on the clinical dose of RA patients and the exchange algorithm of human-mouse body surface area. Mice in the LEF group were intragastrically administered LEF (No.H20080047, Suzhou Changzheng-Xinkai Pharmaceutical Co., Ltd., China) at a dose of 3 mg/kg/d. Mice in the LTD group were intragastrically administered LTD at 27.75 g/kg/d. The normal group and model group were given the same volume of normal saline. Mice were given intragastric administration once a day for 28 consecutive days, and were sacrificed on the 29th day after treatment. The sagittal plane of the radius of the hind limb of the mice was taken every 7 days after the enhanced immunization, and the thickness of the metatarsal joint of the hind limb of the mice was measured with a 50-cell vernier caliper.

### Arthritic Severity Scores of Collagen-Induced Arthritis

The arthritic severity scores of CIA were observed every 7 days after the booster immunization. The specific grading is based on our previously described method (Li et al., 2017). The arthritis score is the sum of all diseased joints of each mouse, and the highest score is 16 points.

### Histopathologic Analysis

The right hind limbs of all mice were taken and placed in 4% paraformaldehyde solution and fixed overnight at 4°C. Next, the excess fur and muscles of the mouse were completely removed, the toe joints were stripped, soaked in 10% EDTA decalcification solution, embedded in paraffin, and cut into 5 μm thickness. These sections were deparaffinized with xylene and rehydrated with gradient ethanol. Then, they were stained with hematoxylin-eosin (HE), Tartrate-resistant acid phosphatase (TRAP), and safranin O fast green (Safranin O) respectively. The stained tissues were observed and photographed using a light microscope (DM RAS2, Leica, Solms, Germany). Histopathological changes in synovial inflammation, cartilage destruction, and bone erosion were assessed and scored according to previously reported methods (Feng et al., 2019). The scores of Loss of safranin O staining were defined as follows (Rauber et al., 2017): no loss (0 score); slight loss (1 score); moderate loss (2 score); severe loss (3 score); complete loss (4 score).

### Flow Cytometric Analysis

The joint synovial tissues were collected from all mice and single-cell suspensions were prepared. Cell Stimulation Cocktail (500X, eBioscience) and Protein Transport inhibitor were added to single-cell suspension cells, strictly following the instructions. The cells were stimulated in a cell incubator at 5%CO2 and 37°C for 5 h. After stimulation, 1 × 106 cells were inoculated into v-type 96-well Microtiter plate preadded with 100 μL labeling buffer, and specific antibodies labeled the antigens on the cell membrane. The eBioscience™ Intracellular Fixation and Permeabilization Buffer Set were applied to fix the membrane of cells. Foxp3 staining buffer set (eBioscience) was used to dilute specific antibodies and tag cytoplasmic and intraconuclear factors. Cells were incubated with the following antibodies: FITC anti-CD4, PE/CY7 anti-IL17A, PE anti-44, PE/CY7 anti-CD25, PE anti-KLRG1, and APC anti-Ki67 (all from BioLegend). Anti-FoxP3 and anti-62L were purchased from eBioscience. Anti-CD127(IL7Ra) and anti-CD278(ICOS) was purchased from miltenyi. 5-Laser/21-Channel Flow Cytometer System (Gallios, Beckman Coulter) was used to detect the number and ratio of relevant cells. The results were analyzed using Beckman’s proprietary software Version1.5.

### Immunofluorescence Analysis

The dewaxed tissue sections were alternately placed in citric acid buffer and heated to boiling point for antigen repair for 20 min, and then returned to room temperature. The cells were permeated with PBS which contained 0.3% Triton X-100 for 20 min. These slices were closed with 3% Donkey serum and incubated in an incubator for 30 min. The specific fluorescent antibody was incubated overnight at 4°C and the fluorescent secondary antibody was incubated for 1 h. Primary antibodies anti-F4/80 antibody (1/100, abcam), anti-iNOS antibody (NOS2) (1/100, abcam), and anti-CD206 (MMR) monoclonal antibody (MR6F3)-PE (1/50, invitrogen) were utilized. For anti-F4/80, anti-iNOS and anti-CD206 staining, donkey anti-rabbit (1/500, Alexa 488,abcam), goat anti-rabbit (1/500, Alexa 647,Invitrogen), and donkey anti-goat (Alexa Fluor^®^555) secondary antibodies were used. After antibody incubation, anti-fluorescence quenching sealing tablets (including DAPI) were added to seal the tablets. Images were collected and analyzed by laser confocal microscopy system (TCS SP8 X, Leica). Immunofluorescent antibody markers were matched with protocols provided in the relevant literature (Yu et al., 2016). F4/80^+^CD206^−^iNOS^+^ cells were M1-type macrophages, and F4/80^+^CD206^+^ iNOS^-^cells were defined as M2-type macrophages.

### ELISA Analysis

The ELISA kit (eBioscience) was used to detect the contents of IFN-γ, IL-17, and IL-4 produced in RA following the instructions strictly.

### Western Blotting Analysis

Proteins for western blot analysis were extracted from the mouse joint synovial tissue and lysed using RIPA Lysis Buffer which contained protease inhibitor cocktail (Applygen Technologies Inc., BeiJing). The protein concentration was quantified preliminarily with the BCA kit. The total proteins were separated using 7.5% SDS-PAGE and then imprinted on NC membrane. They were blocked for 1 h at room temperature using TBST containing 5% skimmed milk. The primary antibodies including anti-mouse Stat-6, Jak-2, Jak-3, phospho-Jak-2, phospho-Jak-3, and phospho-Stat-6 polyclonal antibodies (Abcam, Cambridge, MA, United States) were incubated overnight at 4°C. Next, the membranes were incubated by secondary antibodies (1:2000, Abcam, Cambridge, MA, United States) for 1 h and were treated with ECL chemiluminescence reagents. Three replicates of each experiment were performed. Densitometry plots showing protein expression were analyzed by ImageJ (Bethesda, United States). The housekeeping gene GAPDH was used as an internal reference.

### Statistical Analysis

SPSS (version 20.0) statistical software was used for data analysis. GraphPad Prism (version 8.0) software was used for making graphics. The measurement dates conforming to the normal distribution were expressed as mean ± standard deviation (S.D.). One-way analysis of variance (One-way ANOVA) was used for comparison between multiple groups. For pairwise comparisons between groups, if the variance was uniform, the LSD test was used; if the variance was not uniform, the Tamhane’s T2 (M) test was used. Arthritis scores and plantar joint thickness were evaluated using repeated measures ANOVA. *p* < 0.05 was considered that the difference was statistically significant.

## Results

### Identification of Chemical Constituents in Longteng Decoction

**FIGURE 1:**
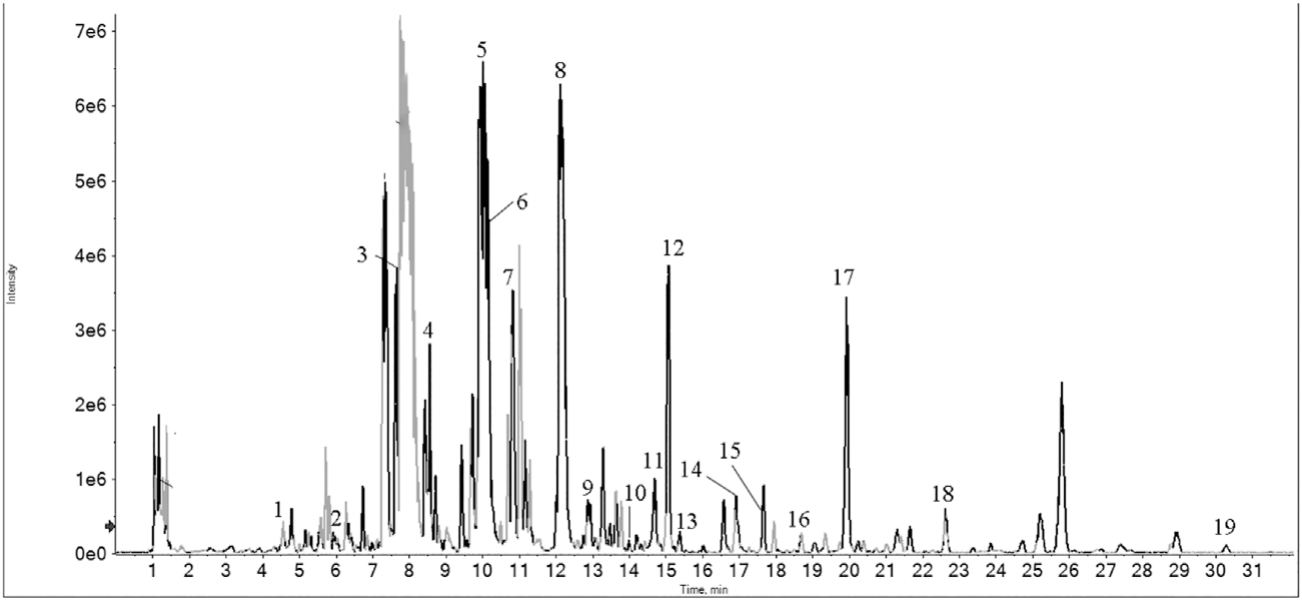
HPLC-ESI/MSn total ion chromatograms of LTD.

A total of 19 main effective constituents in LTD were identified by HPLCESI/MSn. The representative chromatograms and identified compounds are shown in Figure 1 and Table 1.

**TABLE 1:** Chemical components identified from LTD by HPLC-ESI/MSn.

No	Identification	Formula	Duration
1	Piscidic acid	C11H12O7	4.5
2	Oxymatrine	C15H24N2O2	5.97
3	Chlorogenic acid	C16H18O9	7.69
4	Acutumine	C19H24ClNO6	8.4
5	Magnoflorine	C20H24NO4	9.94
6	Albiflorin	C23H28O11	10.21
7	6-Methylcoumarin	C10H8O2	10.82
8	Isoscopoletin	C10H8O4	12.05
9	Paeoniflorin	C23H28O11	12.96
10	Quercetin	C15H10O7	13.96
11	Sinomenine	C19H23NO4	14.61
12	Tracheloside	C27H34O12	15.07
13	Jatrorrhizine	C20H20NO4	15.34
14	Arctiin	C27H34O11	16.91
15	Luteolin	C15H10O6	17.56
16	Artemisinin	C15H22O5	18.68
17	Apigenin	C15H10O5	19.78
18	Arctigenin	C21H24O6	22.63
19	Diosgenin	C27H42O3	30.3

### Longteng Decoction Ameliorates the Severity of Arthritis of Collagen-Induced Arthritis Mice

**FIGURE 2:**
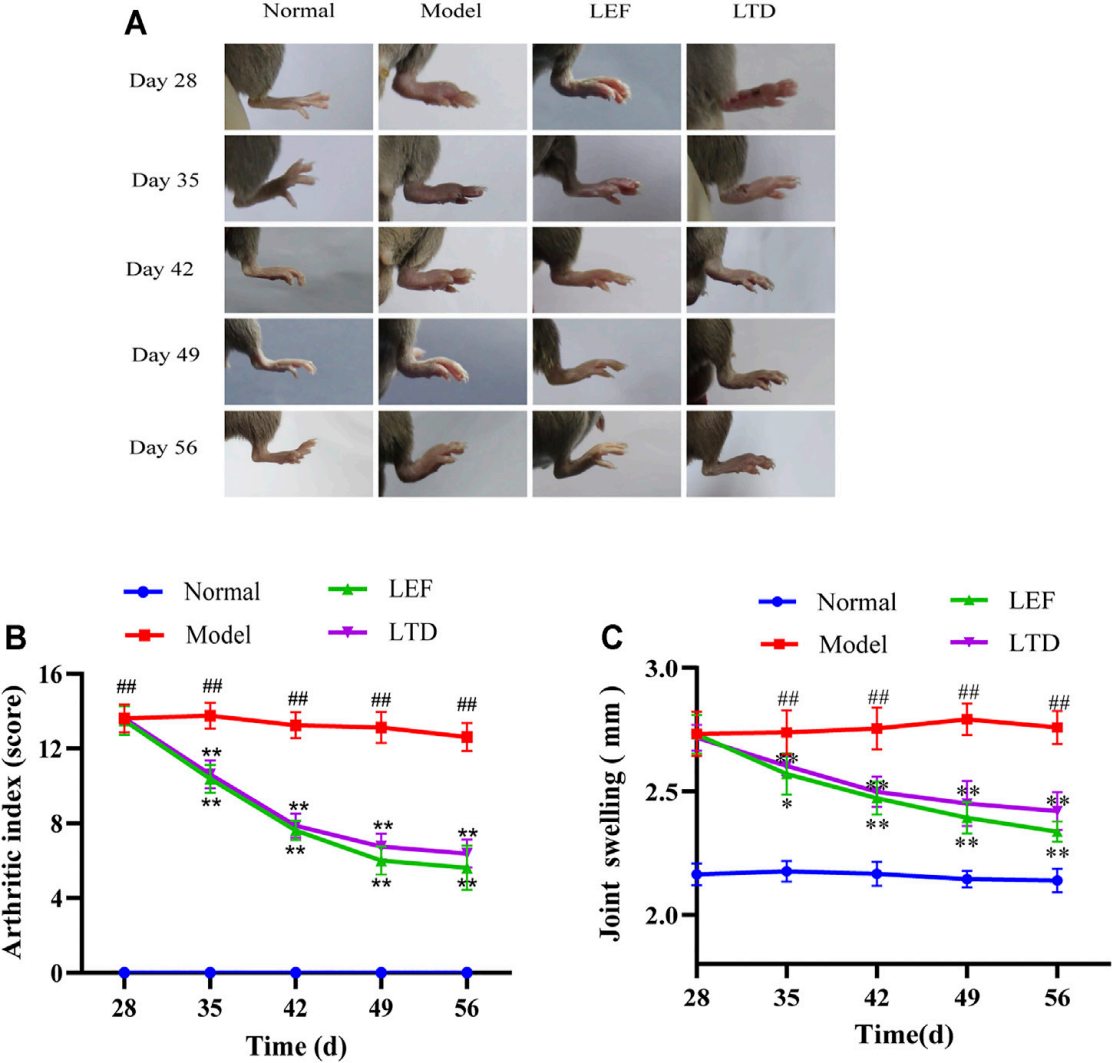
LTD reduced the severity of joint swelling in CIA mice. **(A)** Changes in joint swelling in CIA mice. **(B)** Arthritis index score. **(C)** Changes in thickness of plantar joint in CIA mice. All data are shown as means ± S.D. (*n* = 8); ##*p* < 0.01, compared with the normal group; *p < 0.05, **p < 0.01, compared with the model group.

LTD can relieve joint inflammation in CIA mice and promote the regression of joint inflammation. As shown in Figure 2A, obviously, the redness and swelling of toes and ankles in CIA mice were significantly reduced and the inflammation was relieved after 28 days of treatment with LTD.

In addition, arthritic severity scores of CIA mice were significantly decreased after LTD treatment (Figure 2B). In the arthritis score (Figure 2B), the score of the model group was markedly higher than the normal group. From the 35th day, compared with the model group, the arthritis scores in the LTD and LEF groups were significantly reduced. We also measured the thickness of the bilateral plantar joint in CIA mice throughout the intervention cycle (Figure 2C). The results showed that the swelling thickness of the bilateral plantar joint of mice decreased gradually with the intervention period, and there were significant differences on both sides from the 42nd day.

### Longteng Decoction Ameliorates Histopathology Injury of Joints in Collagen-Induced Arthritis Mice

**FIGURE 3:**
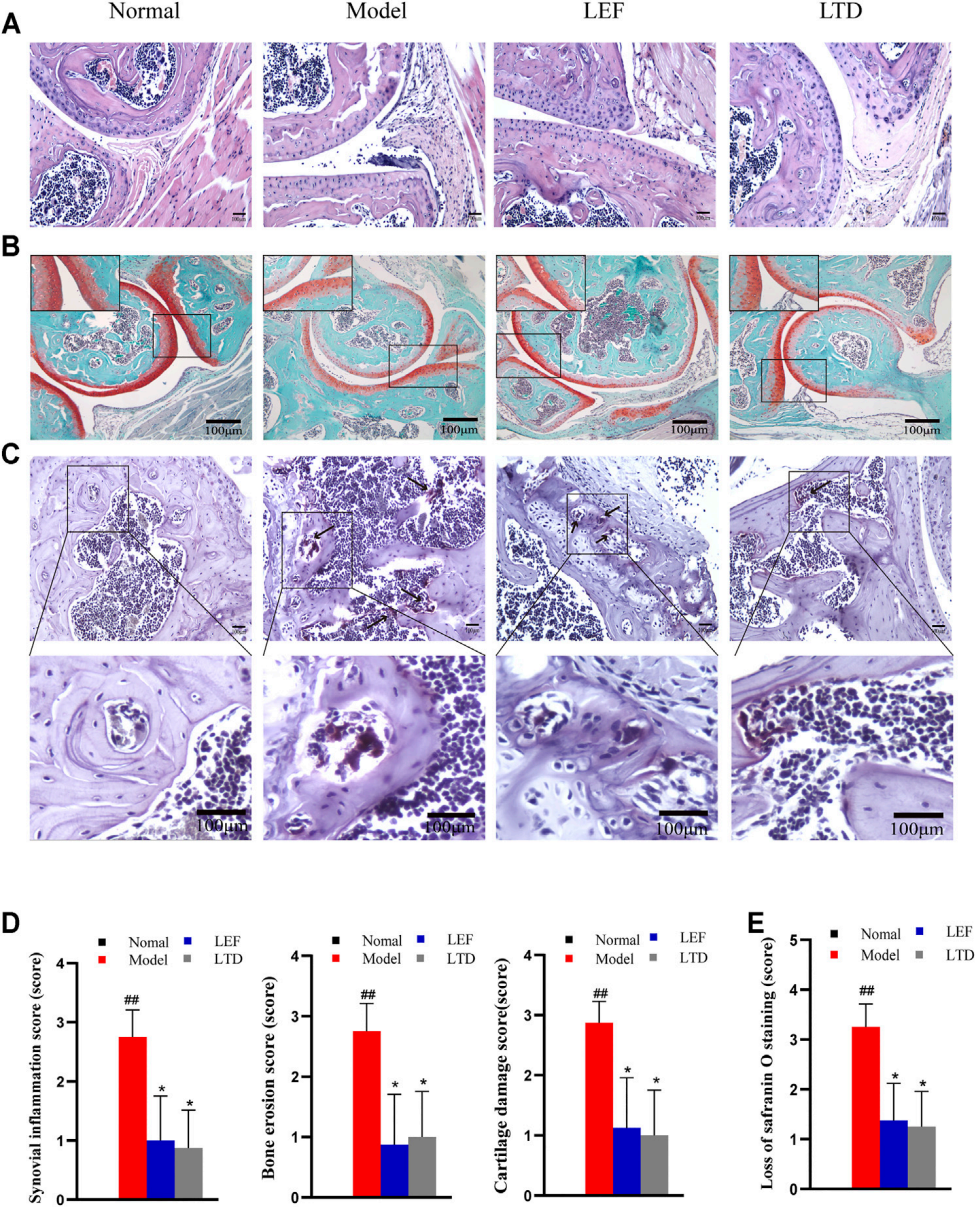
LTD improved histopathological inflammation in CIA mice. **(A)** HE staining of synovial tissue of joint (magnification ×200). **(B)** Saffron O staining of articular tissue (magnification ×100). **(C)** TRAP staining of articular tissue (magnification ×200). The arrow points to osteoclasts. **(D)** Synovial inflammation score, bone erosion score, and cartilage damage score. **(E)** Loss of saffron O staining score. All data are shown as means ± S.D. (*n* = 8); ##*p* < 0.01, compared with the normal group; *p < 0.05 **p < 0.01, compared with the model group. The scale bar corresponds to 100 µm throughout.

There were differences in HE staining among the groups (Figure 3A). In the CIA model group, synovial tissue hyperplasia and massive inflammatory cells infiltration, even multiple cartilage erosion and bone destruction were observed in the joint cavity of mice (Figure 3A). As is shown in Figure 3D, the bone erosion score, synovial inflammation score, and cartilage damage score of CIA mice was notably higher relative to normal mice. Treated with LTD and LEF, the pathological inflammation of HE staining was alleviated and the pathological score was significantly decreased.

In order to further explore the effect of LTD on cartilage destruction in CIA mice, the paraffin sections of mice in each group were stained with Safranin O. As shown in Figure 3B, the staining intensity of CIA mice was observably weaker than that of normal mice. As indicated by the loss of Safranin O staining score (Figure 3E), there was a significant difference between normal mice and CIA mice. After LTD and LEF treatment, the staining intensity was increased and the loss of Safranin O staining score was significantly decreased.

TRAP staining was also performed on paraffin sections of mouse ankle tissue, as shown in Figure 3C. The nucleus is blue-purple and the positive signal is purplish red. Compared with normal group, there were a large number of osteoclasts around the trabecular bone of model group. Compared with model group, these positive signals in all treatment groups were decreased, indicating that the differentiation of osteoclasts was inhibited to varying degrees.

### Longteng Decoction Can Inhibit the Proliferation and Differentiation of Inflammatory Cells

**FIGURE 4:**
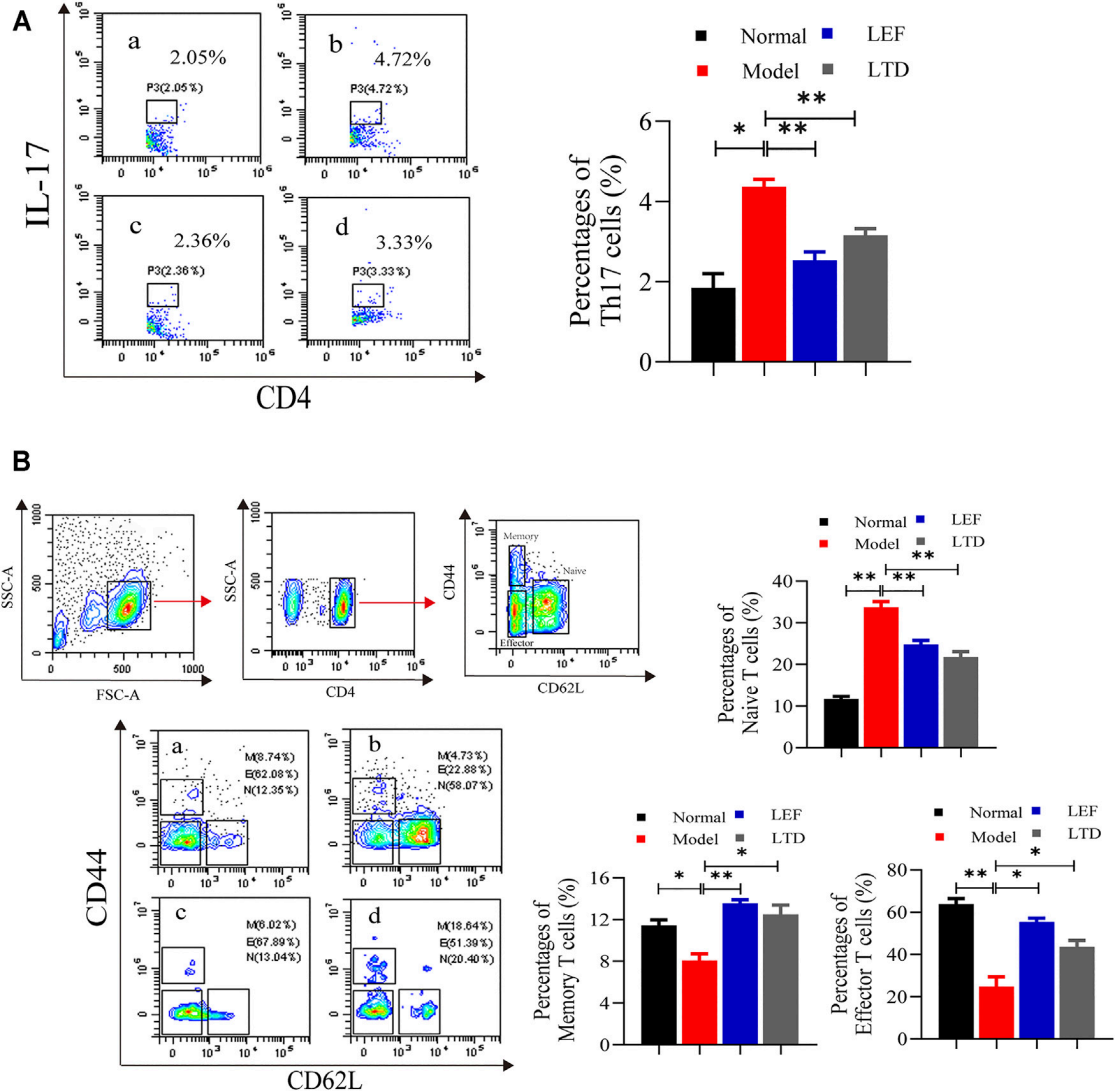
The percentages of Th17, Memory T cells, Naive T cells, and Effector T cells. **(A)** A scatter plot of representative Th17 cells; **(B)** A typical contour map of Memory T cells, Naive T cells, and Effector T cells. The results are presented in the bar charts (*n* = 3). a: normal group, b: model group, c: LEF group, d: LTD group. Data are presented as the means ± S.D.*p < 0.05,**p < 0.01.

It has been shown that human central memory CD4^+^ T cells can secrete IL-17A, IL-21, and TGF-β, through their combination to differentiate naive T into Th17 cells (Li et al., 2008). The percentages of Th17, memory T, naive T, and effector T cells in lymphocytes were detected using flow cytometry. The percentages of Th17 cells (CD4^+^IL-17^+^) of CIA mice were increased significantly, and the abnormal rising of Th17 cells was obviously suppressed after LTD and LEF treatment (Figure 4A). We also observed that the Naive T cells (CD4^+^CD44^−^CD62L^high^) were higher in the model group than in the other groups, while the percentage of effector T cells (CD4^+^CD44^−^CD62L^low^) and memory T cells (CD4^+^CD44^+^CD62L^low^) was lower than LTD and LEF groups (Figure 4B). After LTD and LEF treatment, the percentage of Naive T cells was decreased, and the percentage of effector T cells and memory T cells was increased. The results suggest that LTD treatment can inhibit the proliferation of Th17 cells and Naive T cells.

### Longteng Decoction Can Promote the Proliferation and Differentiation of ILC2s and Treg Cells

**FIGURE 5:**
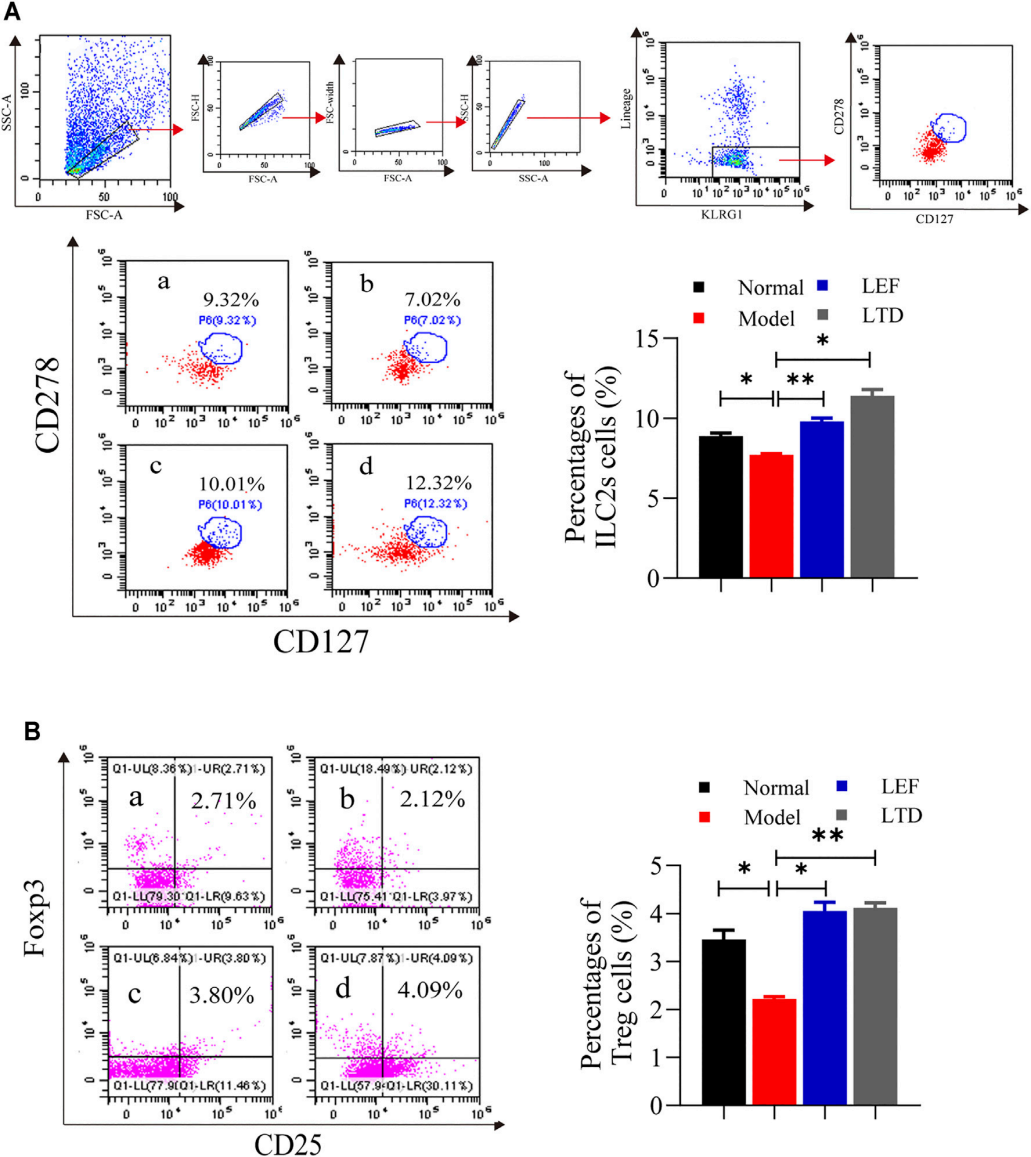
The percentages of ILC2s cells and Treg cells. **(A)** A scatter plot of representative ILC2s cells. **(B)** A scatter plot of representative Treg cells. The results are presented in the bar charts (*n* = 3). a: normal group, b: model group, c: LEF group, d: LTD group. Data are presented as the means ± S.D.*p < 0.05,**p < 0.01.

ILC2s were found in the joint synovial tissues of healthy people. ILC2s can induce Treg cell activation and play an immunomotor role (Rauber et al., 2017). Therefore, the ratio of ILC2s and Treg cells in synovial tissue of the joints were measured by flow cytometry. As shown in Figure 5A, the ratio of ILC2s in the model group was lower than those in the other groups. After LTD and LEF treatment, the ratio of ILC2s was increased. In addition, the ratio of Treg cells (CD4^+^CD25^+^ Foxp3^+^) of the model group were decreased significantly, and the abnormally low levels of Treg cells were significantly increased in the drug intervention groups (Figure 5B). The results suggest that LTD can promote the proliferation of ILC2s and Treg cells.

### Longteng Decoction Can Regulate the Expression Level of JAK-STAT Signaling Pathway Related Proteins

**FIGURE 6:**
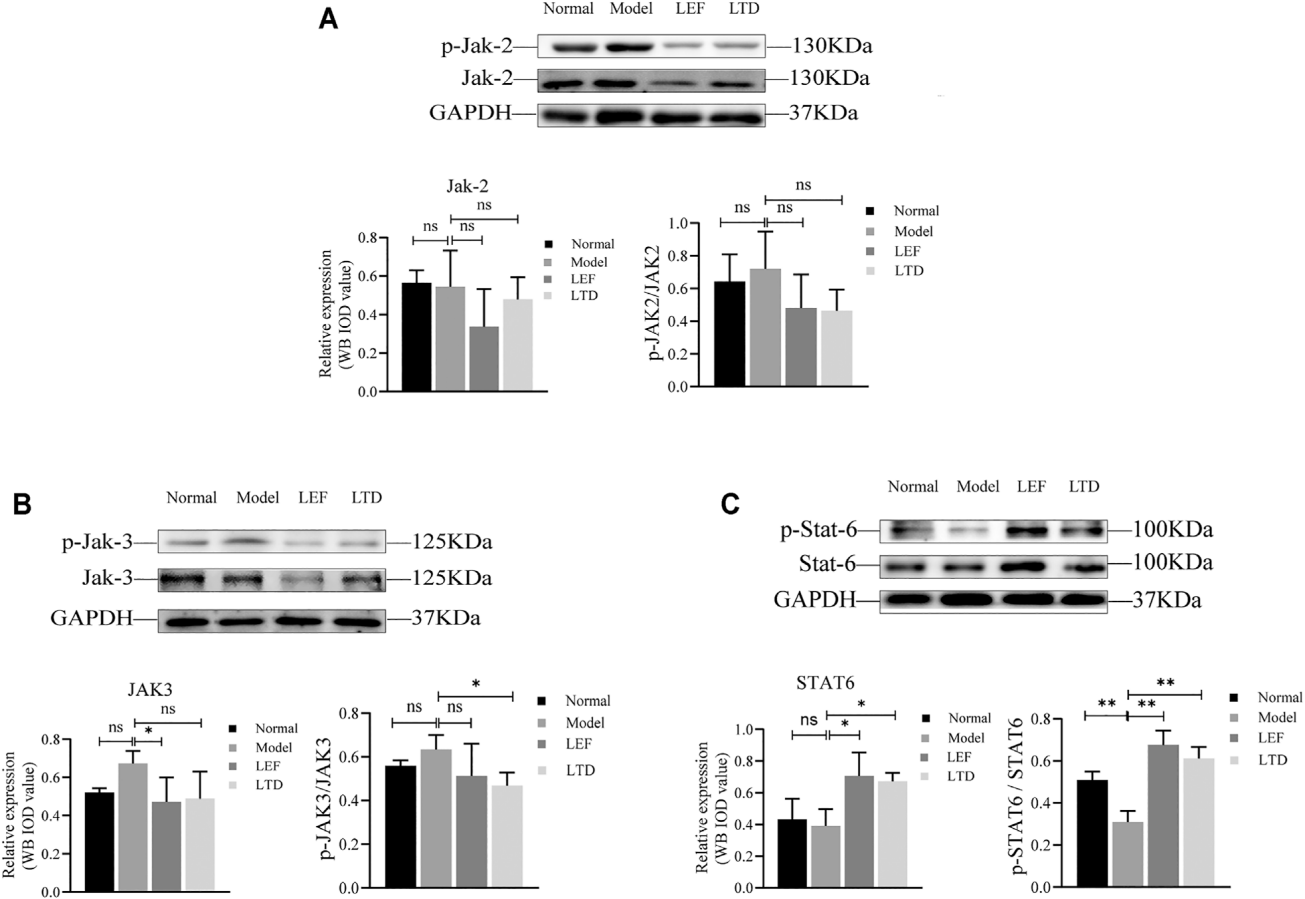
The expression of protein levels. **(A)** The band diagram of Jak2 protein and its phosphorylation level. **(B)** The band diagram of Jak3 protein and its phosphorylation level. **(C)** The band diagram of STAT6 protein and its phosphorylation level. The quantified results are presented in a bar chart (*n* = 3). GAPDH was used as an internal control. Data are presented as means ± S.D. ^ns^ *p* > 0.05, *p < 0.05, **p < 0.01.

We also performed western-blotting to detect the expression and phosphorylation level of key protein molecules in JAK/STAT signaling pathway in synovium of the joint (Figure 6). Surprisingly, our results showed that there was no statistical difference in the expression of JAK2 and JAK3 proteins and their phosphorylation levels after LEF and LTD treatment compared with the model group (Figures 6A,B). Moreover, compared to normal mice, our results also showed that STAT6 protein expression and phosphorylation were both decreased in CIA group while increased markedly in the LEF group and LTD group compared to CIA mice (Figure 6C). These data suggest that LTD inhibits the progression of synovial inflammation in RA by promoting the activation of STAT6 signals, and plays the role of inhibiting pro-inflammatory factors and activating anti-inflammatory factors.

### Longteng Decoction Can Regulate the Secretion of Proinflammatory and Anti-inflammatory Cytokines

**FIGURE 7:**
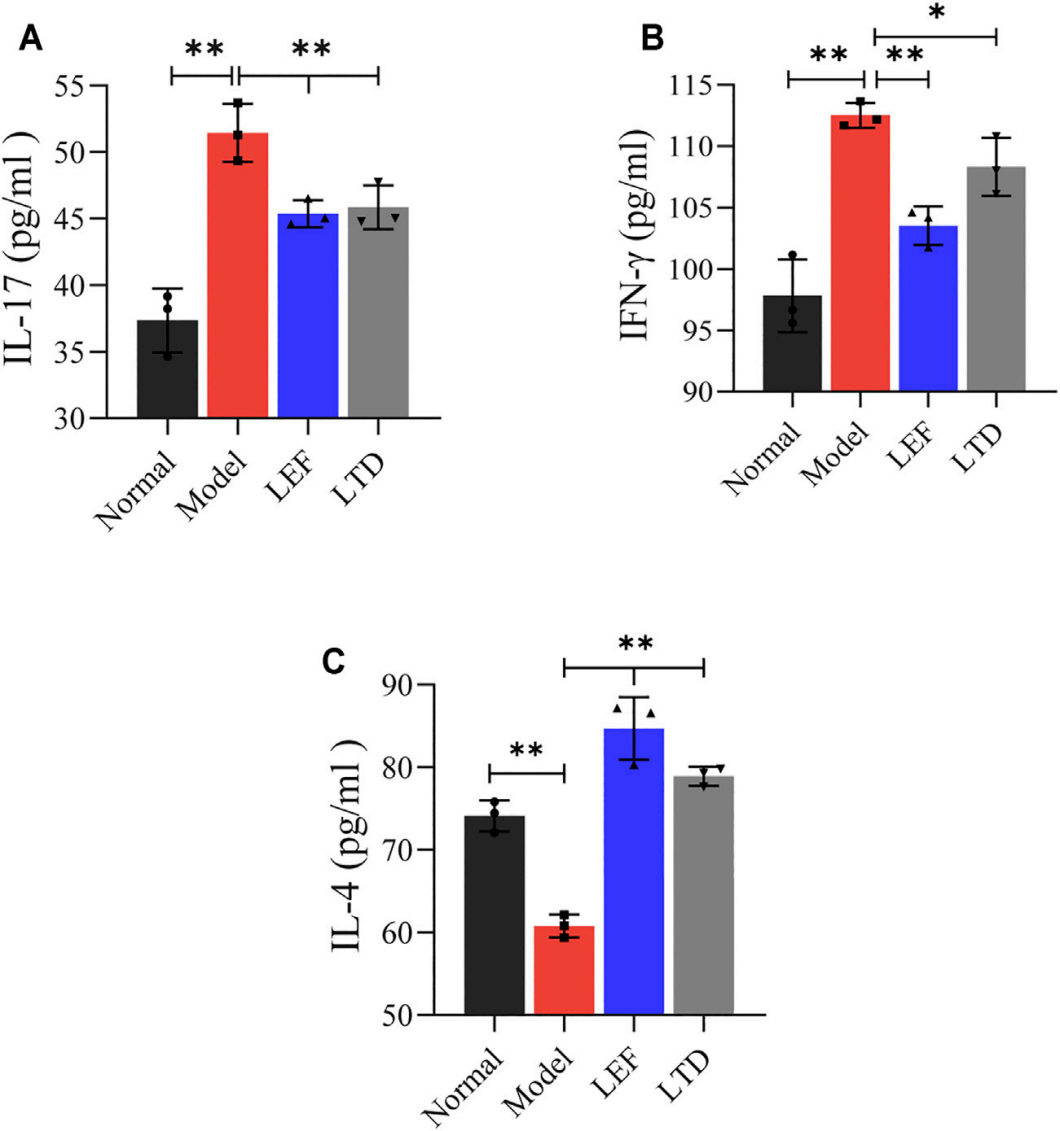
The levels of pro-inflammatory and anti-inflammatory cytokines in serum detected by Elisa. **(A)** The level of IL-17 cytokine. **(B)** The level of INF-γ cytokine. **(C)** The level of IL-4 cytokine. All data are presented as the means ± S.D. (*n* = 3). *p < 0.05 **p < 0.01.

Next, we detected the 3 cytokines associated with RA by Elisa. The levels of predominant pro-inflammatory cytokines associated with our study, including IFN-γ and IL-17, were increased significantly in model groups (Figure 7A,B). After LEF or LTD treatment, the production of these cytokines was inhibited. Moreover, the level of anti-inflammatory cytokine IL-4 was obviously reduced in CIA mice. After LTD treatment, the level of IL-4 was significantly increased (Figure 7C). These data suggest that LTD inhibits the progression of synovial inflammation in RA by inhibiting IL-17 and IFN-γ and promoting the secretion of cytokines IL-4.

### Longteng Decoction Can Promote the Polarization of Anti-inflammatory M2 Macrophages in Synovial Tissue of Collagen-Induced Arthritis Mice

**FIGURE 8:**
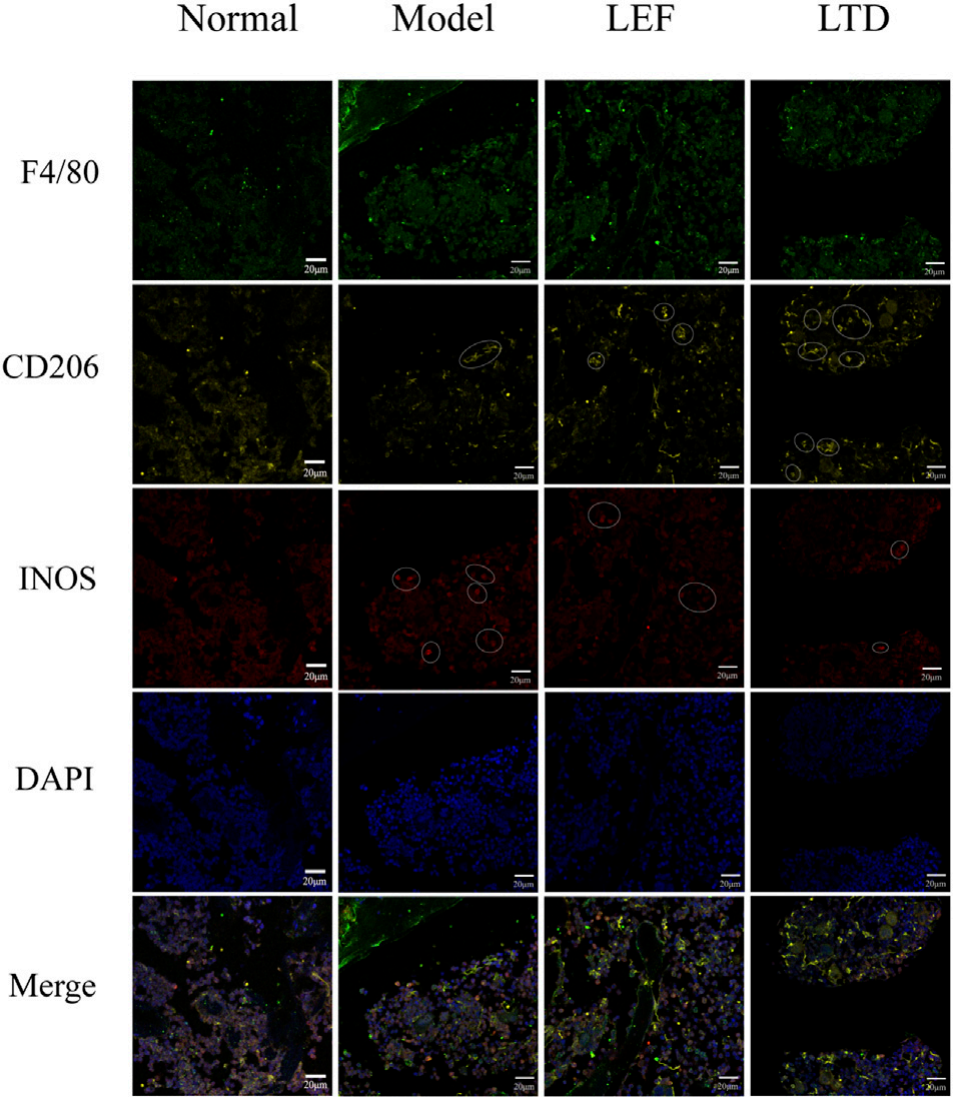
Immunofluorescence images of macrophage distribution. The white circles represent positive cells. The scale bar corresponds to 20 µm throughout.

In order to observe M1 and M2 macrophages in the diseased synovial tissues, immunofluorescence staining was also performed. As shown in Figure 8, expressions of F4/80, iNOS, and CD206 were observed in the pathological tissues of mice. A few positive cells (F4/80^+^, iNOS^+^, and CD206^+^) were observed in the normal group. In CIA model group, F4/80 and iNOS were strongly expressed, and the distribution of macrophages was mainly M1 type. After treatment with LTD, the expression of iNOS in the diseased tissues of mice was decreased compared to CIA model group. While CD206 was gradually increased and the distribution of macrophages was mainly M2 type in LTD group. The results demonstrate that LTD may exert an anti-inflammatory effect by promoting the polarization of M2 macrophages.

## Discussion

RA is a general chronic autoimmune disease which is characterized by synovial inflammation with high morbidity and disability rate (Wang X. et al., 2021). Current treatment strategies for RA mainly focus on targeting pro-inflammatory cytokines and the activation of autoimmune inflammation. Such immunosuppressive therapies often carry the risk of secondary infection and tumor after oversuppression (Lin et al., 2020). Treatment strategies to induce arthritis remission from the perspective of promoting inflammation rather than inhibiting inflammation production have become a new focus of RA treatment research. Innate lymphocytes are a newly discovered group of immune cells. It has similar functions with T cells and can be used as a mediator to connect innate and adaptive immune (Vivier et al., 2018), and has become an important effector cell of the innate immune system (Saferdin and Blüml, 2020). ILC2s play a significant protective part in RA, and adjusting the function of ILC2s is a latent effect target for the therapy of RA (De Pasquale Claudia et al., 2021). Under the guidance of TCM theory, the flexible combination of Chinese herbal medicine and its natural products can prevent and treat patients with diverse syndromes (Li T.-p. et al., 2019). Chinese herbal medicine has long been used as a complementary or alternative medicine to treat RA (Zhang et al., 2018). The combination of Chinese herbs forms a variety of herbal therapeutic formulations, which play a significant synergistic role in treatment (Chen et al., 2020). Our study took this as the starting point to observe the role of the traditional Chinese Medicine Compound LTD in regulating the ILC2s immune response network and slowing down the inflammation of synovial tissue.

As mentioned earlier, the herbal formula for LTD consists of six herbs. Our study identified 19 major chemical components in LTD formulation by HPLC-ESI/MSn analysis. Pharmacological experiment researchers have demonstrated that oxymatrine could inhibit the proliferation of RA synovial cells and regulate the balance of Treg/Th17 (Jiang et al., 2018; Ma et al., 2017). Paeonin can up-regulate FOXO3 by inhibiting Mir-155 expression and prevent FLS proliferation and cytokine release (Liu J. et al., 2017). Scopolamine can inhibit the production of IL-6 by FLS in arthritic rats (Dou et al., 2013), as well as the proliferation and maturation of immature DCs in bone marrow (Wang et al., 2019), playing an anti-RA role. Berberine and apigenin inhibit synovial hyperplasia, angiogenesis, and osteoclast generation through a series of mechanisms that regulate immune responses (Huang et al., 2021; Li et al., 2016; Li Y. et al., 2019). In addition, Isoschaftoside and Jatrorrhizine have been proven to prevent the production of inflammatory mediators (Qiu et al., 2018; Gomes et al., 2016). Pharmacological researchers have also indicated that quercetin can inhibit obvious ameliorate inflammation in CIA mice (Haleagrahara et al., 2017) and inhibit the mechanism of NF-κB activation in arthritis (Yuan et al., 2020), which is a potential medicine for RA. Sinoline plays an anti-inflammatory and immunosuppressive proliferative role by inhibiting angiogenesis and adjusting the secretion of a variety of inflammatory cytokines (Feng et al., 2019; Liu et al., 2018; Huang et al., 2019). Artesunate is a semisyntic derivative of artemisinin, which can inhibit the secretion of TNF-α in RA synovial cells by regulating NF-κB signaling pathway (Li et al., 2013), reduce the activity of IL-17, and regulate the balance of Th17/Treg lymphocytes (Mo et al., 2012; Liu N. et al., 2017). Therefore, we believe that these LTD components can play a synergistic therapeutic effect in promoting the remission and regression of RA inflammation.

The key pathological change of RA is that immune cells continuously infiltrate into the synovial tissues of the affected joints, leading to the occurrence of autoimmune inflammation of the synovial tissues (Firestein and McInnes, 2017). It was found that memory CD4^+^ T cells secreted IL-17A, combined with TGF-β, to differentiate naive T into Th17 cells (Li et al., 2008). In addition, naive T cells can differentiate into effector T and Treg cells (ParkJung et al., 2019). Th17 cells secrete IL-17 to aggravate synovial inflammation (Keiji et al., 2018). The pro-inflammatory cytokines IL-17A and IFN-γ interact synergistically and are involved in the activation of FLS and macrophages, as well as the maturation of osteoclasts, leading to increased cartilage damage (Brian et al., 2015; Lin et al., 2020). Treg cells, on the other hand, produce anti-inflammatory cytokines and inhibit the development of Th17 (Brian et al., 2015) which are indispensable mediators for inhibiting inflammation and maintaining immune tolerance (ParkJung et al., 2019). As a typical anti-inflammatory cytokine, IL-4 can reduce the secreta of IL-17 and inhibit synovial inflammation and bone damage (Mateen et al., 2016). Our experiment also detected a high proportion of Th17 cells and IL-17 and IFN-γin CIA mice. The proportion of Treg cells and the anti-inflammatory cytokine IL-4 was higher in the LTD group. Moreover, the results of HE staining, Safranin O staining, and Trap staining demonstrated that LTD can alleviate the damage of articular cartilage caused by cartilage matrix degradation and loss of proteoglycan, inhibit osteoclast differentiation and synovial inflammatory infiltration, and repair the damage of cartilage and bone. These results indicated that LTD could inhibit the secretion of inflammatory cytokines by down-regulating the proportion of Th17 cells. At the same time, the differentiation of Treg cells was stimulated and the inflammatory progression of RA synovium was inhibited.

The JAK/STAT signaling pathway occupies a vital part in the pathogenesis of RA synovitis (Ptacek et al., 2021). Interactions of pro-inflammatory cytokines can phosphorylate JAK and then recruit and activate STATs proteins involved in signal transduction (Wang et al., 2020). Activation of JAK/STAT signaling pathway stimulates the expression of pro-inflammatory cytokines, induces FLS to proliferate and erodes cartilage in synovium, promotes synovium cell proliferation, and participates in cartilage lesions (Liu et al., 2021). Clinical trials have demonstrated the efficacy of JAK2 and JAK3 inhibitors in the treatment of RA (Wang et al., 2020). STAT6 plays an essential effect in the differentiation process of Treg cells (Sanchez-Guajardo et al., 2007) and can be activated by IL-4 and IL-13 to induce the polarization of macrophages, which is crucial in alleviating the immune activation of arthritis (Chen et al., 2019). Our experiment also observed high expression of STAT6 protein in synovial tissues of mice in the LTD treatment group. Strangely, our current experiments have not been able to prove that LTD has an inhibitory effect on the levels of highly expressed JAK2 and JAK3 proteins. The inhibitory effects of LTD on JAK2 and JAK3 proteins need to be further studied.

Macrophages exert a vital effect in immune response by controlling phagocytosis, producing cytokines, and presenting antigens to naive T cells (Di Benedetto et al., 2019). Macrophages are also significant in the pathogenesis of RA (Ardura Juan et al., 2019). It has been found that compared with healthy joints, there are more macrophages in the synovial tissue of inflammatory RA (Mulherin et al., 1996; Tak et al., 1997). These cells are the primary source of cytokines, chemokines (such as CCL2 and CXCL8), and degrading enzymes that lead to synovial proliferation and ultimately to cartilage and bone destruction (Edilova et al., 2020). Macrophages are divided into two different phenotypes, M1 and M2, and play different roles. M1-type macrophages can be activated by IFN-γ and TNF, and can also secrete IL-23 to induce the production of Th17 and IL-17, which has a pro-inflammatory effect (Siouti and Andreakos, 2019). In the process of inflammatory injury, synovial cells release IL-33, which specifically induces the differentiation of Th2 cells and the transformation of macrophages into an anti-inflammatory M2 phenotype (Schmitz et al., 2005). Activated M2 macrophages release IL-4, IL-13, IL-10, and TGFβ, inhibit pro-inflammatory cytokines and the activation of osteoclasts, and participate in the regression of inflammation, angiogenesis, and tissue remodeling and repair (Tardito et al., 2019). It was demonstrated that F4/80^+^CD11b^−^ macrophages increased when the disease subsides, and their polarization is in favor of anti-inflammatory M2 macrophages (Tu et al., 2019).

In this experiment, our dates show that LTD treatment can regulate the STAT6 protein expression and promote the transformation of M1-type macrophages into the anti-inflammatory M2 phenotype. It also helps to regulate the secretion of inflammatory cytokines and shows immunosuppressive activity, thus slowing down the inflammatory process of synovial tissue and accelerating the regression of joint inflammation.

It was reported that ILC2s were reduced in the blood and synovial biopsy of RA patients (Leijten Emmerik et al., 2015). Our study also found a decrease in the percentage of IC2s cells in synovial tissue in CIA mice compared to the normal group. ILC2s is characterized by high expression of GATA-3 and production of Th 2 cytokines (IL-4) (Ebihara, 2020). After treatment with LTD, the number of ILC2s in the pathological tissues of CIA mice increased significantly. By secreting IL-4 and IL-13, ILC2s can inhibit or slow down the pathological changes of synovial inflammation in RA joints and play the immune regulatory function (Yasunori et al., 2018). It can also activate the STAT6 signaling pathway (Chen et al., 2019), promote the differentiation of M2 macrophages through the autocrine growth factor IL-9, release TGF-β, induce the recruitment and activation of Treg cells, inhibit the proliferation of Th17 cells and the secretion of pro-inflammatory cytokine IL-17, and improve inflammatory arthritis (Rauber et al., 2017).

In our study, LTD proved to be an effective herbal formula for treating RA. It can reduce the inflammatory swelling of joints in CIA mice, inhibit inflammation infiltration of synovial tissue, and relieve the cartilage damage. The main mechanisms may be related to regulating the proliferation of ILC2s and Th cells, regulating the activation of JAK/STAT signaling pathway, promoting the transformation of M1-type macrophages into anti-inflammatory M2 phenotype, inhibiting the secretion of pro-inflammatory factors, and restoring the homeostasis of RA tissue environment. These results of this research provide a basis for further LTD supplementation in the treatment of autoimmune arthritis including RA. Taken together, our results suggest that LTD can be considered as a supplement or replacement to traditional medicines in the treatment of RA.

## Data Availability

The original contributions presented in the study are included in the article/Supplementary Material, and further inquiries can be directed to the corresponding authors.
